# Language Dependency of /s/ Production: Native Dutch Versus Non-Native English

**DOI:** 10.1177/00238309241242114

**Published:** 2024-04-20

**Authors:** Meike M. de Boer, Willemijn F. L. Heeren

**Affiliations:** Leiden University Centre for Linguistics, Leiden University, The Netherlands

**Keywords:** Forensic speech science, fricatives, multilingualism, language dependency, within-speaker consistency

## Abstract

With forensic recordings being collected in multiple languages increasingly often, this study investigates the language dependency of the voiceless alveolar fricative /s/ in speakers of native (L1) Dutch and non-native (L2) English. Due to phonetic similarity between the languages, Dutch learners of English may exhibit language-independent /s/ acoustics, making it an interesting feature for multilingual forensic speaker comparisons (FSCs). However, the findings show that out of the four spectral moments, center of gravity, standard deviation (*SD*), skewness, and kurtosis, only *SD* remained stable across the languages; the other measurements were language-dependent. The results were largely independent of the /s/ tokens’ contexts, although an interaction between language and context was found for skewness and kurtosis: With a labial right phonetic neighbor, language dependency was largely reduced. The findings have implications for FSCs: as /s/ is language-dependent in speakers of L1 Dutch and L2 English, it shows limited potential for cross-linguistic speaker comparisons in forensic casework.

## 1 Introduction

The voiceless alveolar fricative /s/ has been described as rather speaker-specific (e.g., Kavanagh, 2012; Schindler & Draxler, 2013; Van den Heuvel, 1996). Speaker-specificity refers to relatively low within-speaker variation and high between-speaker variation. This allows for speaker classification using /s/ as a feature—for example, in the context of law enforcement. So far, most forensically-motivated research on speaker-specificity has concerned monolingual contexts (cf. Bradlow et al., 2017; Künzel, 2013; Lo, 2020; Mok et al., 2015), and cross-linguistic investigations on case materials are not recommended by the International Association for Forensic Phonetics and Acoustics (IAFPA, 2020). However, as speech evidence in real casework may involve speech material in more than one language (Campbell et al., 2004; Künzel, 2013; Van der Vloed et al., 2014), practice requires the exploration of speech parameters that remain consistent within individual speakers, across their languages.

Speech sounds, or segments, could be language-*in*dependent if they are acoustically similar or (near-)identical in two languages. If a segment is produced with the same articulatory settings in two languages, a speaker does not have to make any adaptations and their realization may be consistent across their first (L1) and second language (L2). Also, if there are subtle differences that are not easily perceived, for instance, because the acoustic differences do not reflect a difference in meaning in the L1, an L2 speaker may transfer their L1 acoustics to the L2 (Flege, 1995). In both cases, the segments’ spectral parameters are expected to be language-independent within the speaker and could potentially be used for cross-linguistic speaker comparisons. In prior work, we investigated language dependency of the bilabial nasal /m/ (De Boer & Heeren, 2023), a segment with the same acoustic settings in L1 Dutch and L1 English, and of the hesitation markers *uh* and *um* (De Boer & Heeren, 2020), of which the vowel is acoustically similar but not identical in the two languages. Results showed that although neither case showed “perfect” L1 transfer, some acoustic parameters remained consistent across the L1 and L2. For /ə/ in *uh* and *um*, there was a cross-linguistic difference in the first and second formants, but not in the third formant or fundamental frequency (De Boer & Heeren, 2020). For /m/, we found that nasal formants (N1−N4) and their bandwidths overall remained consistent across speakers’ L1 and L2, although some individuals showed small between-language differences (De Boer & Heeren, 2023).

For this study, we investigated language dependency of the sibilant /s/ among the same speaker group, with L1 Dutch and L2 English. The Dutch and English /s/ have distinct acoustics, with Dutch /s/ often described as somewhere in between English /s/ and /ʃ/ (e.g., Quené et al., 2017). However, as this contrast between Dutch and English /s/ is potentially not picked up by Dutch learners of English, this pair may form an example of a sound that is phonetically similar but not identical (cf. Quené et al., 2017). Although prior work by Quené et al. (2017) found language dependency among the same student population as studied here, we investigated if this result would hold in speech that is more representative of that found in forensic casework. In addition, as /s/ is often described solely by its center of gravity (CoG), this study provides a more thorough understanding of the spectral characteristics of /s/ in L1 Dutch compared with that in L2 English.

### 1.1 Speaker-specificity of /s/

Fricatives are the consonants most often considered in forensic casework (Gold & French, 2011), of which /s/ is thought to have the most potential for discriminating between speakers (Schindler & Draxler, 2013; Van den Heuvel, 1996). During the production of /s/, a constriction is made between the tongue and the alveolar ridge. This directs the airstream toward the upper teeth, generating turbulent noise (Stuart-Smith, 2007, p. 66). Between-speaker differences in /s/ acoustics arise due to the size and shape of the cavities on both sides of the constriction, which depend not just on the anatomical characteristics of the speaker but also on the constriction’s exact location and on the shape of the tongue (Kavanagh, 2012, pp. 58–59).

Although /s/ consistently performs among the best-discriminating segments, or at least consonants, findings regarding the speaker-specificity of /s/ when compared with other speech segments are somewhat contradictory. Van den Heuvel (1996) compared the speaker-specificity of several vowels, nasals, and /s/ in Dutch, and found that /s/ performed less well than the other segments. In contrast, Kavanagh (2012) found that together with nasals, /s/ is among the best-performing consonants for speaker classifications in British English. She found this result mainly for the first two spectral moments, but also to a lesser extent for the third and fourth (see Section 2.2 for an explanation of the spectral moments). Similarly, Schindler and Draxler (2013) reported that in spontaneous speech in German, /s/ showed the highest speaker-specificity of all consonants and vowels in their study, including nasals and other fricatives. Their results showed that all of the fricative’s spectral moments carry approximately equal speaker-specific information.

### 1.2 Within-speaker consistency of /s/

For a feature to be speaker-specific, there must be a decent amount of within-speaker consistency, here meaning that a speaker shows relatively stable /s/ acoustics across contexts and settings. For /s/, this consistency has been hypothesized to hold across languages as well. Quené et al. (2017) describe Dutch and English /s/ as an example of non-identical but phonetically similar sounds (p. 519). According to Flege’s (1995) Speech Learning Model, L2 sounds that are similar to a sound in a speaker’s L1 are most difficult to fully acquire, because learners merge the sounds into one phonetic category. Hence, they transfer the acoustic parameters from their L1 rather than adopting a native-like pronunciation. Indeed, Dutch and English /s/ are acoustically very similar. However, when compared with English /s/, Dutch /s/ is produced with a slightly more retracted and flatter tongue and more lip rounding, resulting in a lower CoG (Collins & Mees, 2003; Quené et al., 2017).

When empirically testing the language dependency of /s/, Quené et al. (2017) found that their L1 Dutch speakers made a contrast in /s/ acoustics between L1 Dutch and L2 English, with a higher CoG in L2 English. Hence, they concluded that the L2 speakers in their sample, who had above-average L2 proficiency, learned the difference between Dutch and English /s/. However, the question may not have been answered fully, due to several choices made in their original study. First, a set of 25 speakers only was used, among whom were both males and females. Second, the speech tasks were not only spontaneous but included read speech as well, which cannot be considered representative for a speaker’s natural, conversational speaking style (Nakamura et al., 2008). Third, /s/ tokens were sampled from specific phonetic contexts only, that is, Dutch and English homophones, thus ignoring any contextual variation in the majority of tokens in the dataset and not allowing for a natural distribution of /s/ contexts as would occur in spontaneous speech. Although Quené et al. (2017) were able to show that the speakers distinguished between the Dutch and English /s/, we think that language independency in spontaneous materials has not been ruled out and deserves further investigation.

Although prior work has shown that taking into account all contexts rather than selecting tokens from particular contexts increases speaker discrimination (e.g., Schindler & Draxler, 2013; Smorenburg & Heeren, 2020), it is also known that /s/ acoustics depend on the immediate phonetic context due to coarticulation. Anticipatory labialization has a lowering effect on fricatives’ CoG, because the anterior cavity is lengthened by the forward lip movement. This effect has been found for right phonetic neighbors requiring lip rounding (e.g., /u, w/), but also lip closure (e.g., /p/; Bell-Berti & Harris, 1979; Koenig et al., 2013; Munson, 2004). This means that in a multilingual study, between-language differences may be found due to a different context in which /s/ typically occurs, rather than a language-dependent articulation.

### 1.3 Research question and predictions

This study elaborates on the study of Quené et al. (2017), who studied cross-linguistic differences in /s/ among the same student population and found that speakers had different /s/ acoustics in L2 English than in L1 Dutch. Our study investigates whether this language dependency remains after making several adjustments to the study design. We used a larger and more homogeneous speaker sample, selected spontaneous speech only, and sampled all available /s/ tokens regardless of their phonetic context. In addition, as Schindler and Draxler (2013) found speaker-specific information in all four spectral moments, we included them all in the current analysis. To take phonetic context effects into consideration, which may differ between English and Dutch and hence can alter any language effect found, the main focus of this study is on the interaction between language and phonetic context. This study investigated to what extent speakers differ in their /s/ acoustics between L1 Dutch and L2 English spontaneous speech when phonetic context is taken into account.

Based on the findings by Quené et al. (2017), despite the changes in our design we still expect a cross-linguistic CoG difference, where the CoG is higher in L2 English than L1 Dutch. As a labial acoustic neighbor following /s/ also showed to have a lowering effect on CoG (Munson, 2004), if such contexts are overrepresented in one language as compared with the other, we may find an interaction effect between language and context. In addition, /s/ tokens with a labial right neighbor have been shown to have a lower second spectral moment, the spectral standard deviation (*SD*; Smorenburg & Heeren, 2020), which may again cause an interaction effect when contexts are not distributed equally in both languages. Although skewness and kurtosis give a more complete overview of the spectrum (Kavanagh, 2012) and have been found to contribute to speaker-specificity in German (Schindler & Draxler, 2013), there are no known between-language or context effects to predict any (interaction) effects. Hence, skewness and kurtosis may be language-independent within speakers, and *SD* may remain consistent if there are no major differences in typical contexts of /s/ between Dutch and English.

## 2 Method

### 2.1 Materials and procedure

Recordings were selected from the Database of the Longitudinal Utrecht Collection of English accents (D-LUCEA;^1^ Orr & Quené, 2017). This database contains speech data of students from University College Utrecht (UCU), of whom most speak English as an L2 and were also recorded in their L1. As about 60% of the UCU student population is Dutch (Quené et al., 2017), we focus on speakers of L1 Dutch and L2 English. After some reading tasks, the speakers gave a two-minute informal monologue in L1 Dutch and then in L2 English to an interlocutor who was proficient in both languages.^2^ In the monologues, students spoke about small talk topics of their own choice (e.g., hobbies and vacations). Participants were informed about the monologues during recruitment and some had prepared their monologues or selected a topic beforehand. Overall, the monologues can be considered (semi-)spontaneous (cf. Orr & Quené, 2017).

We selected 53 female speakers with L1 Dutch, as this was the largest homogeneous subgroup in the database. Speakers who spoke another native language besides Dutch, had a salient regional accent in Dutch, or were recorded after 1 month after their arrival at UCU were excluded, as Quené et al. (2014) showed that over time, convergence takes place in the English spoken as lingua franca at UCU. The students had just started their first year of higher education and were between 17 and 20 years old (*M* = 18.4, *SD* = 0.8). In the Netherlands, English is taught at primary and high school from about age 11. As L2 English proficiency is one of the selection criteria to be admitted to UCU, the speakers in the database have an above-average L2 proficiency (Quené et al., 2017).

The speakers were recorded using a close-talking microphone attached to a headset (Sennheiser HSP 2ew). Further details about the recording sessions are described by Quené et al. (2017).

### 2.2 Segmentation and measurements

/s/ tokens were identified automatically with the help of hand-made orthographic annotations and were manually validated by at least two coders in Praat (Boersma & Weenink, 2023). Tokens that were distorted or shorter than 30 ms were excluded from the analysis (cf. Jongman, 1989). For each /s/ token, the directly adjacent phonetic neighbors were transcribed manually and then characterized as labial or non-labial (cf. Smorenburg & Heeren, 2020). Labial contexts included rounded vowels /u, ʊ, ɔ, o, ø, y, Y/ and consonants /w/, (partially) rounded diphthongs /œy, ɑu/, and bilabial consonants /p, b, m/. All other contexts, including silent pauses and labiodental consonants, were considered to be non-labial.^3^ Table 1 shows the number of tokens per context per language.

**Table 1.** Number of /s/ Tokens per Context (Non-Labial, Labial) and Language (Dutch: 2,399; English: 2,432).

Context	Dutch (L1)		English (L2)	
	Left	Right	Left	Right
Non-labial	2,193	2,033	2,212	1,855
Labial	206	366	220	577

The four spectral moments CoG, *SD*, skewness, and kurtosis were measured using Praat (Boersma & Weenink, 2023). These parameters were selected as they are often used to reflect the spectrum of fricatives (Kavanagh, 2012) and have been found to be speaker-specific for /s/ (Kavanagh, 2012; Schindler & Draxler, 2013). The CoG is also referred to as the spectral mean (Stuart-Smith et al., 2003, p. 1852). The second spectral moment gives the *SD* of the spectrum, that is, the dispersion around CoG (Kavanagh, 2012). Finally, skewness and kurtosis represent the degree of symmetry and peakiness of the energy distribution in the spectrum, respectively (Jongman et al., 2000, p. 1253).

Measurements were done over the middle 50% of /s/ tokens, over the 550−8,000 Hz band, using power spectrum weighting. This pre-analysis filtering was applied to reduce the effects of partial voicing, for example, due to coarticulation. Previous studies have shown that /s/ in Dutch and a variety of (non-)Western languages has CoGs not exceeding 6,000 Hz (Gordon et al., 2002; Ditewig et al., 2021; Quené et al., 2017), so we do not expect to lose much information above 8,000 Hz.^4^ Values > 2 *SD* from the by-language means were considered outliers and treated as missing data. For CoG, there were 64 outliers in Dutch and 55 in English, for *SD*, there were 132 in Dutch and 158 in English, for skewness, there were 83 in Dutch and 88 in English, and for kurtosis, there were 64 in Dutch and 87 in English.^5^ The measurements can be downloaded from the Supplemental Materials.

### 2.3 Statistical analysis

The data were analyzed using linear mixed-effects models in *R* (R Core Team, 2021) with the *lmer*() function from package lme4 (Bates et al., 2015). Fixed factors were Language (L1 Dutch, L2 English), LeftContext (non-labial, labial), and RightContext (non-labial, labial) and were treatment-coded with L1 and non-labial as reference levels. As this study focuses on a possible interaction between language and the tokens’ left and right context, models were evaluated through likelihood ratio testing with manual backward stepwise exclusion of predictors, starting from a maximal main model including the two interactions between Language and (left and right) Context. The optimal model was the simplest model that did not show a significant change in fit when compared with the more complex model. Finally, random slopes for Language over Speaker were added to investigate how speakers varied in the extent to which they showed between-language differences.^6^ For all models, likelihood ratio testing showed that these random slopes contributed to the optimal model, that is, that there was by-speaker variation in the Language effect.

The spectral moments showed weak to moderate correlations (see Table 2) but as together they reflect the spectrum best (e.g., Kavanagh, 2012; Schindler & Draxler, 2013), they were all included in the analysis. As we built four separate models (i.e., for each of the spectral moments), a Bonferroni correction was used (α = 0.0125).

**Table 2.** Correlation Matrix of the Four Spectral Moments for (a) All Tokens in the Dataset, and (b) for Each of the Two Languages Separately.

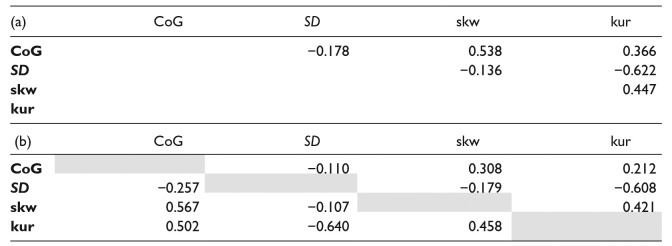

Note: In (b), the Top Right Corner (Right From/Above the Gray Cells) Gives the Correlations in L1 Dutch; the Lower Left Corner (Left From/Below the Gray Cells) Gives those in L2 English. All Correlations Were Significant (*p* < .01).

## 3 Results

An overview of the means and *SD*s of the four spectral moments of /s/ (i.e., CoG, *SD*, skewness, and kurtosis) can be found in Table 3. For neither CoG nor *SD* were interaction effects included in the optimal model, showing that any language effect that is found is independent of context (see Table 4). For CoG, the model including two interaction effects between Language and Context did not perform better than a model with just one, χ^2^(1) = 4.16, *p* = .04 (for Lang × LeftContext), χ^2^(1) = 1.57, *p* = .21 (for Lang × RightContext). The optimal model was a simpler model including just the main effects. In English, CoG was more than 800 Hz higher than in Dutch, showing a substantial between-language difference. Moreover, a labial left or right phonetic neighbor had a lowering effect on CoG. Table 4 gives an overview of the fixed parts of the optimal model.

**Table 3.** Mean (and Standard Deviation) of the Four Spectral Moments of /s/ in L1 Dutch (*n* = 2,113) and L2 English (*n* = 2,086).

	L1 Dutch	L2 English
**CoG (Hz)**	5,047 (829)	5,812 (772)
***SD* (Hz)**	1,256 (262)	1,251 (283)
**Skewness**	0.82 (0.59)	1.17 (0.73)
**Kurtosis**	4.51 (4.28)	5.44 (4.33)

**Table 4.** Optimal Linear Mixed-Effects Models Predicting the CoG and *SD* of /s/ in Dutch (L1) and English (L2). Reference Levels: Dutch, Non-Labial.

	CoG (Hz)		*SD* (Hz)	
	Coefficient (*SE*)	*t*	Coefficient (*SE*)	*t*
Intercept	5,137.25 (74.62)	68.85	1,248.53 (18.11)	68.95
language = English	807.90 (54.18)	14.91		
leftContext = Labial	−235.33 (31.45)	−7.48		
rightContext = Labial	−316.76 (22.75)	−13.92		

**Figure 1.**
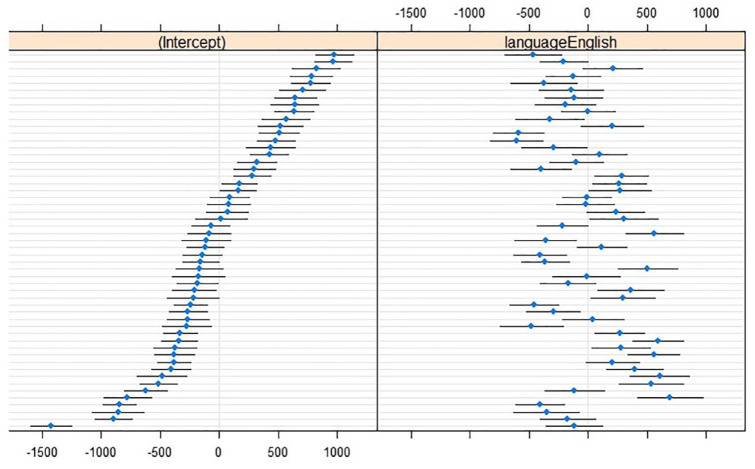
Caterpillar plots of CoG (in hertz; reference level L1 Dutch) with (left) by-speaker intercepts relative to the cross-speaker intercept (*x* = 0) and (right) by-speaker effects relative to the main effect of Language (*x* = 0). Horizontal lines represent 95% confidence intervals for individual speakers, sorted from lowest to highest CoG intercept in L1 Dutch.

Random slopes of Language over Speaker contributed to the optimal CoG model, χ^2^(2) = 290.24, *p* < .001; some speakers made larger between-language adaptations than others (see Figure 1).

**Figure 2.**
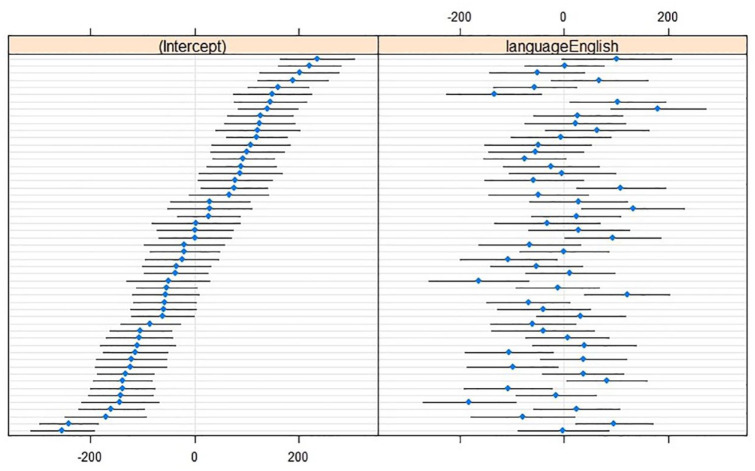
Caterpillar plots of *SD* (in hertz; reference level L1 Dutch) with (left) by-speaker intercepts relative to the cross-speaker intercept (*x* = 0) and (right) by-speaker effects relative to the main effect of Language (*x* = 0).

For *SD*, the most complex model did not outperform a model with only one interaction effect, χ^2^(1) = 1.21, *p* = .27 (Lang × LeftContext); χ^2^(1) = 0.04, *p* = .84 (Lang × RightContext). Further simplification showed that the optimal model did not contain any interaction or main effects. Adding random slopes for Language over Speaker improved the model, χ^2^(2) = 77.29, *p* < .001, showing that although there was no overall effect of language, individual speakers may differ in their *SD* across their L1 and L2 (see Figure 2).

**Figure 3.**
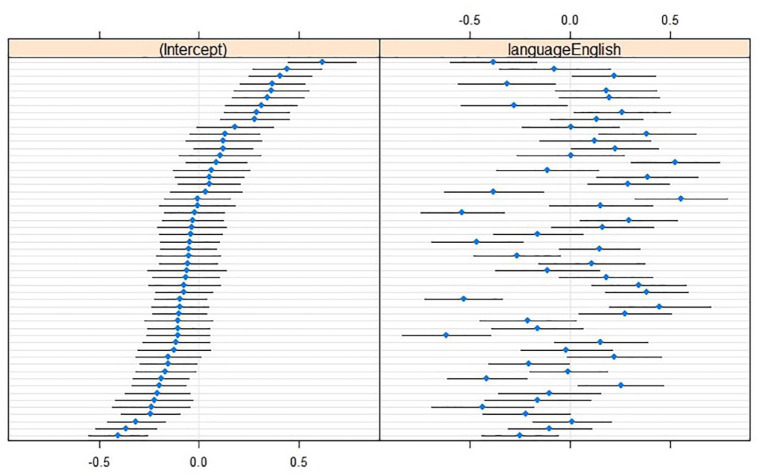
Caterpillar plots of skewness (in hertz; reference level L1 Dutch) with (left) by-speaker intercepts relative to the cross-speaker intercept (*x* = 0), and (right) by-speaker effects relative to the main effect of Language (*x* = 0).

For skewness, a model with a Language × RightContext interaction effect, including LeftContext as a main effect, was as good as the maximal model, χ^2^(1) = 0.61, *p* = .44. In fact, it turned out to be the optimal model because it performed better than a model without the inclusion of LeftContext, χ^2^(1) = 16.38, *p* < .001, or with just main effects, χ^2^(1) = 21.92, *p* < .001. Skewness was more positive in L2 English, and somewhat less positive near a labial left neighbor. When the right neighbor was labial, the language effect decreased and the skewness was more similar between the languages (see Table 5). Adding random by-speaker slopes for language contributed to the model, χ^2^(2) = 219.69, *p* < .001 (see Figure 3).

**Table 5.** Optimal Linear Mixed-Effects Models Predicting the Skewness and Kurtosis of /s/ in Dutch (L1) and English (L2). Reference Levels: Dutch, Non-Labial.

	Skw (Hz)		Kur (Hz)	
	Coefficient (*SE*)	t	Coefficient (*SE*)	t
Intercept	0.85 (0.03)	26.36	4.55 (0.25)	18.10
language = English	0.41 (0.05)	8.86	1.18 (0.28)	4.17
leftContext = Labial	−0.12 (0.03)	−3.96		
rightContext = Labial	−0.01 (0.03)	−0.24	0.44 (0.24)	1.81
lang = Eng × right = Labial	−0.21 (0.04)	−4.68	−1.17 (0.31)	−3.73

**Figure 4.**
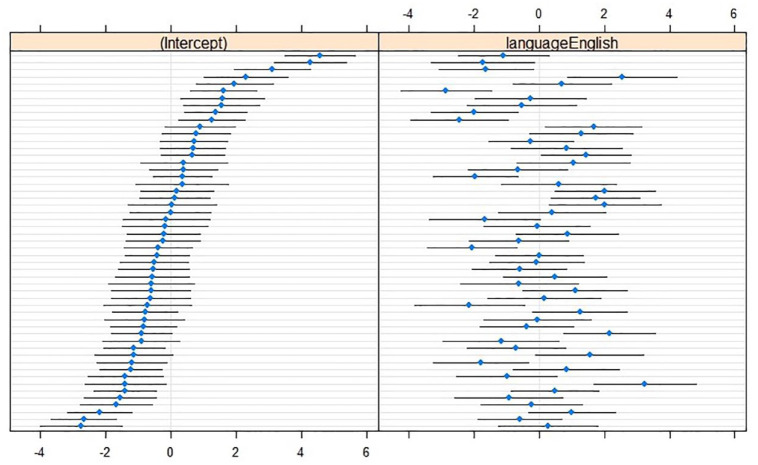
Caterpillar plots of kurtosis (in hertz; reference level L1 Dutch) with (left) by-speaker intercepts relative to the cross-speaker intercept (*x* = 0), and (right) by-speaker effects relative to the main effect of Language (*x* = 0).

The optimal model for kurtosis included the Language × RightContext interaction, but no interaction or main effect with LeftContext. In L2 English, kurtosis was more peaked than in L1 Dutch in non-labial contexts. However, when RightContext was labial, this effect was almost entirely neutralized (see Table 5). Random slopes for language were included in the optimal model, χ^2^(2) = 131.9, *p* < .001 (see Figure 4).

## 4 Discussion and conclusion

This study investigated the possible language independency of acoustic parameters of /s/ in a group of speakers with L1 Dutch and L2 English. Our findings show that out of the four spectral moments, which all have speaker-specific characteristics (Schindler & Draxler, 2013), only *SD* remained stable across the speakers’ languages. As in previous work, speakers differentiated between their /s/ realization in L1 Dutch and L2 English: CoG was higher in English than in Dutch (cf. Quené et al., 2017). The lack of an interaction effect showed that this between-language difference was not context-dependent, although CoG was lower with a labial phonetic neighbor. Skewness and kurtosis, which were not considered in prior work, were language-dependent as well.

Quené et al. (2017) studied a similar question among the same student population to see if the phonetic difference between the Dutch and English /s/ was acquired by the students. The goal of this study was to investigate if /s/ could be to some extent language-independent, and hence could be useful in forensic speaker comparisons (FSCs) involving multilingual recordings. We considered the study by Quené et al. (2017) unfit to answer our research question due to several design choices. First, the study sampled only 25 students from the database, including both male and female speakers. Second, besides spontaneous speech, read speech was included, which is acoustically different from conversational speech and thus not representative of speech found in forensic settings (Nakamura et al., 2008). Third, only /s/ tokens from Dutch-English homophones were selected, which ignored many tokens and reduced the naturalness of the /s/ tokens’ distribution. Finally, Quené et al. (2017) only investigated the CoG, which gives an incomplete view of the spectrum. Hence, in our study, we doubled the sample size and selected female speakers only to create a homogeneous group; we segmented /s/ tokens from spontaneous, conversational speech only; we selected all available /s/ tokens regardless of their context—while controlling for context in our models; and we analyzed all four spectral moments (see Kavanagh, 2012; Schindler & Draxler, 2013).

As was found by Quené et al. (2017), studying 25 male and female students from the same population, CoG of /s/ was higher in L2 English than in L1 Dutch. The between-language difference we found was 808 Hz, which is smaller than the difference of 1,373 Hz reported by Quené et al. Thus, by including more speakers from a homogeneous group with just female speakers, and by considering all /s/ tokens in spontaneous speech only, we found a less substantial difference in cross-linguistic /s/ realization. When looking at the composition of the sample, the decreased between-language difference is somewhat surprising, as female speakers are generally described as more proficient in language learning and use more standard pronunciations (e.g., Huang, 2023; Xia, 2013). If that would be the case, we would expect a females-only sample to make a clearer differentiation between their L1 and L2 /s/ than a sample including male speakers. At the same time, the students at UCU are selected based on their L2 English proficiency and the male speakers in the sample could be very proficient as well. In addition, Quené et al. (2017) did not find any gender differences in their study. Hence, the different sample composition does not seem to cause the decreased between-language difference. An alternative explanation is that between-language differences are smaller in spontaneous than in read speech, as spontaneous speech shows spectral reduction when compared with read speech (Nakamura et al., 2008); this may result in less pronounced differences. Despite finding a smaller between-language difference, it was still substantial and corroborates Quené et al. (2017)’s conclusion that L1 Dutch speakers with above-average L2 English proficiency have language-dependent /s/ realizations.

Similar to CoG, skewness and kurtosis were language-dependent, with the distributions in L2 English being more tailed and peaky than in L1 Dutch. However, an interaction effect showed that the difference between L1 Dutch and L2 English halved or even disappeared when the right phonetic neighbor was labial. As prior literature shows, the right phonetic neighbor has a larger effect on /s/ acoustics due to anticipatory articulation (e.g., Bell-Berti & Harris, 1979; Koenig et al., 2013; Munson, 2004). When /s/ is followed by a labial sound, this may lead to more similar /s/ acoustics regardless of the language spoken. Although the parameters in this study correlated somewhat, the pair with the highest correlation, kurtosis and *SD*, yielded different results: Kurtosis was language-dependent and *SD* was not.

The only feature remaining stable across L1 Dutch and L2 English was the second spectral moment, *SD*. Hence, although the spectral mean (CoG) shifts across languages, the dispersion of energy around this mean (*SD*) remains stable. *SD* also seemed to be context-independent, which is partly in line with Koenig et al. (2013) who found that the *SD* of /s/ tokens in a labial context was higher than in a non-labial context, but that this effect was smaller than context effects on the other spectral moments. This shows that of the features considered here, *SD* may be the most suitable for FSCs including speech material in both Dutch and English. However, the use of merely one feature reduces discriminatory power; excluding CoG from a classification model of Dutch /s/ reduced speaker classification accuracy from 19.5% to 13.9% (cf. Smorenburg & Heeren, 2020). This shows that *SD* alone may not be powerful enough to make /s/ a useful segment for cross-linguistic FSCs. In addition, random slopes showed that speakers behaved differently in their adaptations across their languages: Some speakers had higher SDs in their L2, some lower, and some remained (almost) consistent. Hence, the language independency found for *SD* across speakers is somewhat unstable and unpredictable for individual speakers.

To summarize, although the cross-linguistic difference found in this study was smaller than that found by Quené et al. (2017), /s/ realizations still turned out to be language-dependent in speakers of L1 Dutch and L2 English. Hence, based on our results, we do not consider the set of spectral moments of /s/ to be suitable features for FSCs involving more than one language.

## Supplemental Material

sj-csv-1-las-10.1177_00238309241242114 – Supplemental material for Language Dependency of /s/ Production: Native Dutch Versus Non-Native English Supplemental material, sj-csv-1-las-10.1177_00238309241242114 for Language Dependency of /s/ Production: Native Dutch Versus Non-Native English by Meike M. de Boer and Willemijn F. L. Heeren in Language and Speech

## Notes

1. The data can be obtained through CLARIN-NL: https://dev.clarin.nl/node/4183.

2. For two students in the sample, the order of the Dutch and English informal monologues was reversed.

3. Labio-dental consonants do not involve lip rounding or closure and were therefore not considered labial (cf. Smorenburg & Heeren, 2020).

4. For the data used here, in L1 Dutch the mean spectral peak for /s/ was 4,918 Hz (*SD* = 1,304 Hz), and in L2 English, the mean was 5,943 Hz (*SD* = 1,278 Hz). This shows that with cutting the frequencies above 8,000 Hz, we kept the majority of information.

5. The analysis was replicated without all tokens with at least one outlying parameter (*n* = 632). Other than marginally different estimates, this did not lead to different results. The more complete models are presented in this article.

6. Left and right phonetic neighbors were not included in the random parts of the models because they are not the main focus of this study and based on prior literature, by-speaker variation in context effects is expected to be minimal. A test evaluating models where by-speaker slopes for leftPhon and rightPhon were included (regardless of their contribution to the model) only led to marginally different model estimates.
